# Adjacent Vehicle Number-Triggered Adaptive Transmission for V2V Communications

**DOI:** 10.3390/s18030755

**Published:** 2018-03-02

**Authors:** Yiqiao Wei, Jingjun Chen, Seung-Hoon Hwang

**Affiliations:** Division of Electronics and Electrical Engineering, Dongguk University-Seoul, Seoul 04620, Korea; weiyiqiao@dongguk.edu (Y.W.); jjchen@dongguk.edu (J.C.)

**Keywords:** vehicle communication, vehicle-to-vehicle communications, data transmission method, adaptive modulation scheme

## Abstract

For vehicle-to-vehicle (V2V) communication, such issues as continuity and reliability still have to be solved. Specifically, it is necessary to consider a more scalable physical layer due to the high-speed mobility of vehicles and the complex channel environment. Adaptive transmission has been adapted in channel-dependent scheduling. However, it has been neglected with regards to the physical topology changes in the vehicle network. In this paper, we propose a physical topology-triggered adaptive transmission scheme which adjusts the data rate between vehicles according to the number of connectable vehicles nearby. Also, we investigate the performance of the proposed method using computer simulations and compare it with the conventional methods. The numerical results show that the proposed method can provide more continuous and reliable data transmission for V2V communications.

## 1. Introduction

Recently, vehicular communication has been attracted as a crucial use case for 5G communication. The vehicles can communicate directly with other vehicles or adjacent infrastructures, such as access points (APs), or fixed equipment beside the road referred to as roadside units (RSU) [1]. The vehicular ad-hoc network (VANET), a variation of the mobile ad-hoc network (MANET), is a technology used for an intelligent transport system (ITS) where the moving vehicles are wirelessly connected [2]. The initial motivation and major goal for the VANET are to improve road safety, increase transit efficiency, and scale down the impact of shipping on the environment [3]. In the meantime, other kinds of applications rapidly grow due to the passengers’ desire for IP-based Internet services such as internet surfing, email, file download, multimedia entertainment, parking, and tourist guide information. These require the introduction of wireless local area network (WLAN) technology to the VANET [4], such as IEEE 802.11p or short-range communication (DSRC) since 2010 [5]. The concept of drive-thru systems was suggested in [6], where each RSU AP connects to the internet cloud server and the vehicles moving in the AP’s coverage can connect to the AP. Then, through the RSU AP, the vehicles can acquire IP-based internet connection and a variety of infotainment services. However, due to the high mobility of vehicles, the sparse deployment of RSU APs, and the limited capacity of the AP, it is hard to guarantee the quality and stability of communication between the vehicle and the AP. On the other hand, vehicle-to-vehicle (V2V) communication can take Wi-Fi direct technology. The Wi-Fi direct is a standard defined by the Wi-Fi Alliance to enable devices to directly communicate with others [7]. Therefore, in [8], an easily installed inter-vehicle communication system utilizing the Wi-Fi direct was proposed and verified for the VANET scenario. Additionally, since the IEEE 802.11p has been the sole standard technology realizing the V2V until now [9], the Wi-Fi direct can easily be incorporated with the IEEE 802.11 standards [10].

The V2V has potential to be an alternative method to enhance the on-road internet service in real-life situations, such as platoons or traffic jams. Platooning refers to vehicles moving in a convoy with a short distance, which can be a promising technology with advantages of higher traffic throughput and higher energy efficiency [11]. For the V2V communication, the platoon or the traffic has an identical channel characteristic, which has a specular component and low Doppler shift, since the vehicles are closely spaced and relatively static. Since it is a relatively optimistic channel situation, V2V communication can be considered as an assistant method to bridge the service to the vehicles. More detailed cases are necessary to be investigated for the vehicular communication [12]. Here, are two challenges in platooning and traffic jam scenarios.

• The vehicles in the gap area between the APs’ coverage

**Figure 1:**
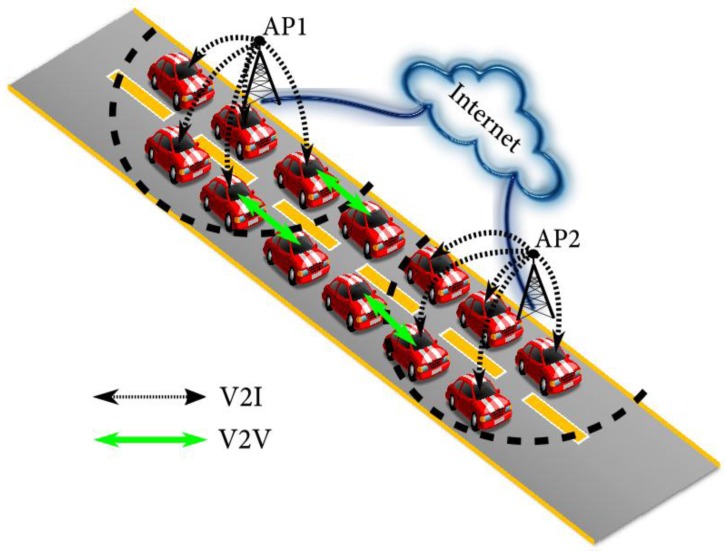
The vehicles in the gap area between the APs’ coverage.

Due to the sparse AP deployment, this may happen due to the limited coverage of the APs. In this case, it is difficult for the users in vehicles to achieve reliable and stable internet service. In the Figure 1, the vehicles are located in the gap area between the coverages of the AP1 and the AP2, where the vehicles are hard to connect to the APs when, for example, the drivers want to download the tourist information through the electric map. The feasible solution is to acquire the data from the adjacent vehicle in the AP coverage. This backup solution can be approached as a V2V relay, where the AP coverage can be extended and, thus, the area spectral efficiency be improved [13]. In [13], an approach using channel state information was proposed to increase the reliability of the V2V relay. In [4,14], two efficient opportunistic relay protocols were proposed.

• Data traffic jam in traffic jams or platoons

**Figure 2:**
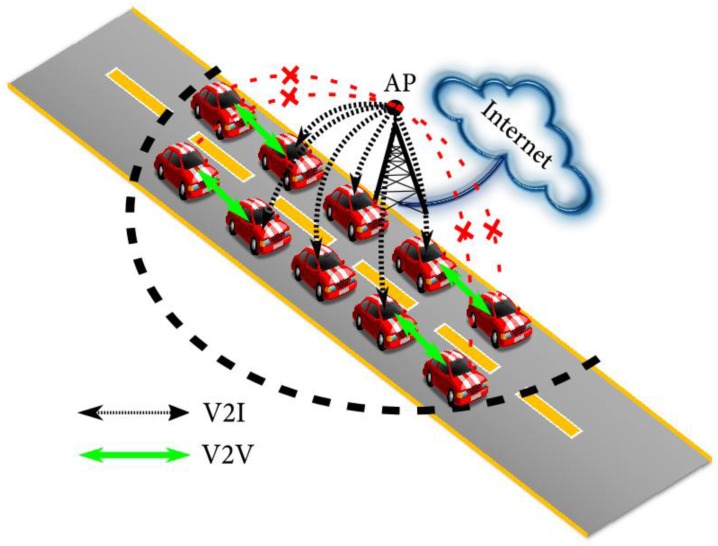
Data traffic jam in traffic jams or platoons.

In a platoon or a traffic jam, the quality of wireless service is difficult to be guaranteed if the service is only delivered through the V2I. We can expect that lots of passengers in platooning vehicles want to enjoy the internet services such as multimedia streaming with high data traffic demand [15]. Particularly, when the location of the vehicles in the traffic jam is not well covered by the service area, the users will suffer from the low service quality. In Figure 2, where many cars under the traffic jam belong to only one AP, huge service demand is requested by the vehicles which may exceed the maximum capability for the single AP. Therefore, some vehicles may not acquire the service from the AP. The solution can be that the vehicles which can acquire the data from the AP by connecting to the cloud server will store it and disseminate it to other vehicles. In [16], a similar case was examined, where the V2V was exploited to assist the V2I to deliver advertisements from the network to the vehicles. The results indicated that the exploitation of the V2V enhanced the information dissemination in the vehicular network when RSU deployment was sparse or dense [16].

From the examples, it is obvious that the enhancement of the unreliable service delivery in the V2V networks is required for the future. Furthermore, the V2V still faces some challenges such as a harsh communication environment due to the severe scattering without line-of-sight and the frequent topology changes of the VANET due to the high mobility of vehicles [17].

Therefore, in this paper, we propose an adaptive data rate selection scheme according to the changes of physical network topology in order to improve the reliability and continuity of data transmission in V2V communications. Focusing on the physical layer aspects, a new algorithm is suggested, and its performance is investigated in terms of error probability and throughput. The paper is organized as follows: In Section 2, we review the related literature. Our proposed method is presented in Section 3. In Section 4, the simulation results are analyzed. Finally, our findings and further work are described in Section 5 of the conclusion.

## 2. Related Works

According to the performance analysis in [18], the stability and reliability of IEEE 802.11p based vehicle communication are difficult to be guaranteed. The primary reason is that IEEE 802.11p uses the same physical layer as the IEEE 802.11a standard, which was originally designed for a relatively stable environment like an indoor environment [18]. In the 802.11p standard, with the combination of four types modulation (BPSK, QPSK, 16QAM, and 64QAM) and three kinds of coding rate (1/2, 2/3, and 3/4), the data rate varies from 3 Mbps to 27 Mbps. Although various data rate choices are available, a conventional rate adaption (RA) algorithm is used, in which only one type of data rate will be selected by transmitter vehicle according to the channel condition estimation before transmission starts [19]. Some early research regarding rate selection also fixed the data rate to the highest option [20] or the lowest [21]. In order to overcome the challenges resulting from the mobility of vehicles and the variation in transmission situation, the scalability of the physical layer (PHY) must be improved, as discussed in [22]. Adaptive modulation and coding (AMC) is a technology to be introduced into a long term evolution (LTE) system to maximize the throughput of the system by changing the modulation and coding method adaptively, according to the current channel situation [23]. Following the same idea, some researchers have already made efforts in improving the transmission scheme of the PHY. In [24], a novel approach called automatic doppler shift adaptation (ADSA) is proposed to compensate for the doppler effect by extensive simulation. Also, a loss differentiation rate adaption scheme, which can choose the optimal data rate by taking interference losses and fading losses to enhance the reliability of V2V communication, is designed in [19].

Conventional RA schemes, such as AMC and the literatures mentioned above, all focus most of their attention on the channel condition but do not take variations of network topology into consideration when designing scalable transmission schemes. In order to acquire high throughput and high-reliability V2V transmission, the time-varying topological structure of the vehicular network is also a serious issue needing to be addressed even if the channel situation is good enough. Specifically, consider a situation in which vehicles are moving as a platoon on the road. In this platoon, one vehicle may decide to depart the platoon for some reason, then the connection will be broken if some other vehicle is communicating with it. Although the transmitter vehicle can choose to temporally store the data received from APs into a buffer and transmit it later when receiver vehicles appear, the transmission speed may not be high enough to compensate for the drop of throughput caused by the interruption, especially when the transmission speed is fixed to a low data rate. On the other hand, always fixing the data rate to quite a high level is arbitrary because, in V2V scenario channels, quality is not always optimal.

For the purpose of addressing the issues described above, in this work, we propose a physical topology adapting transmission scheme in which a vehicle can adjust the transmission data rate by changing the modulation type according to the number of the nearby connectable vehicles. The innovation of this scheme is using a scalable physical layer to handle the degradation of system performance caused by unpredictable changes in physical topology. As far as we know, there is no other research or physical device which is similar to the proposed physical topology triggered adaptive data transmission scheme in the field of transport or other areas of application. Through the simulation results, this adaptive scheme is verified to be able to improve the performance of the V2V communication transmission.

## 3. Adaptive Transmission Scheme for V2V Based on Physical Topology

In this section, the proposed adaptive transmission scheme is described. When the number of available connected vehicles is high, the V2V communication may be resumed with another neighboring vehicle, even if the current connection is broken. Therefore, the data transmission can be kept more continuous and reliable since the data stored in the buffer can be transmitted even with a low data rate. On the other hand, if the number of connectable vehicles is low, it is hard for the vehicle to resume the transmission since there are few chances to link to another vehicle. Therefore, it is necessary to transmit the information stored in the buffer in a more aggressive manner, with a higher data rate. Here, we utilize the beacon signal between the vehicles as an auxiliary means to achieve the dynamic adjustment of the data rate according to the random topology changes. In the vehicular communication systems, the beacon signal plays a special role in providing rich information such as location, heading and other status information [25]. We assume the vehicles are equipped with the periodic broadcast beacons to announce its presence to the surrounding vehicles. For simplicity and clear demonstration of the proposed algorithm, the following assumptions are made: the channel and position information are integrated in the beacon signal, its exchange between vehicles is frequent enough, the vehicle can detect the number of adjacent connectable vehicles when receiving the beacons, and the algorithm is operated for the vehicle (called a data donor) which transmits the data to other vehicles.

**Figure 3:**
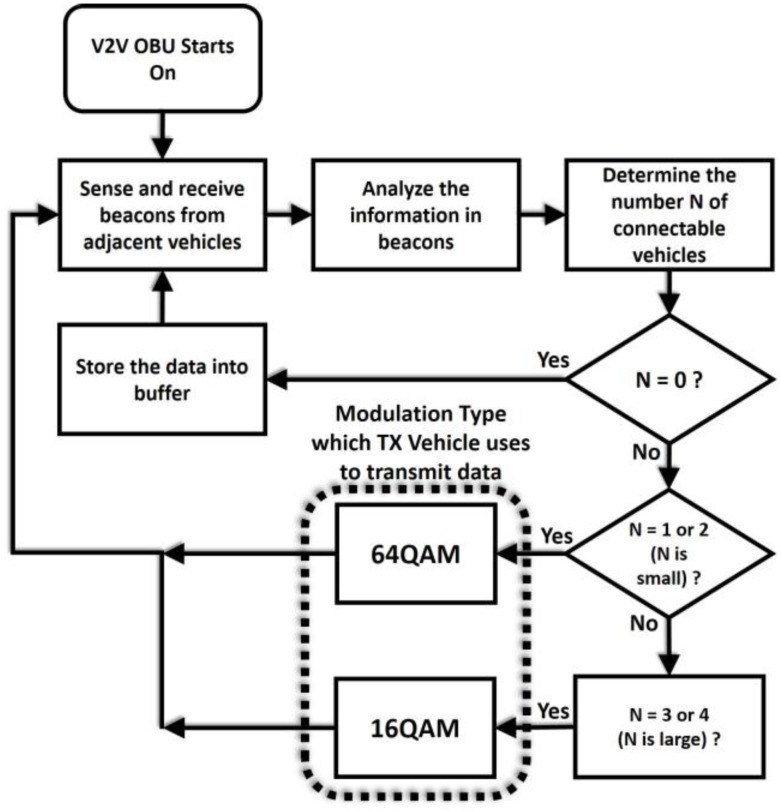
Flow chart for the proposed algorithm.

Figure 3 shows the flow chart of the proposed algorithm. Once the vehicle starts, it keeps sensing the beacon signal broadcasted by adjacent vehicles and determines the number of connectable vehicles denoted as N. When N equals to zero, the vehicle stores the data in the buffer without transmission, since there are no adjacent vehicles. When N is equal to a number larger than zero, the communication can be established. However, if the connection is broken due to a vehicle leaving the platoon, it seems difficult to connect to another vehicle again. In order to minimize the possible data loss when no adjacent vehicle exists, a more aggressive data rate selection can be considered.

We try to reason why the possible rates are chosen and describe the details as follows. To guarantee the reliability of the data transmission, i.e., to keep the bit error rate (BER) lower than the threshold, the received power at the vehicle *P_R_* must be larger or equal to the receiver sensitivity *S_R_* [26]. The *S_R_* is usually defined for a different threshold value for different modulation and coding types, as seen in Table 1 [27]. As the table shows, a higher level modulation requires a higher *S_R_* and, thus, asks for a higher received power. *P_R_* can be calculated as

(1) $$P_{R}=P_{T}+G_{T}+G_{R}-L-I-F$$

where *P_T_* is the transmitter power of the transmitter, *G_T_* is the transmitter antenna gain, *G_R_* is the receiver antenna gain, *L* is the path loss, *I* is the interference, and *F* is the channel fading. We assume that the *P_T_*, *G_T_*, and *G_R_* values are fixed. When there are fewer vehicles around, the interference and the channel fading is reduced, since less scattering causes better channel conditions due to less adjacent vehicles [28]. Therefore, a sufficient *P_R_* can allow for the achievement of values higher or equal to *S_R_* (such as 64QAM). Meanwhile, when the number of vehicles is large, the interference and the channel fading become higher. Therefore, a lower *P_R_* cannot allow for a value higher or equal to *S_R_* to be chosen. Therefore, a lower order modulation is chosen. Therefore, a higher order modulation such as 64QAM is selected because the number of connectable vehicles is low. Meanwhile, as N increases, there are more and more connectable vehicles which can be backup options. Therefore, the data rate can be adjusted to be lower (16QAM) so as to ensure the reliable communication in the harsh channel condition. With the proposed algorithm, more reliable and continuous data transmission for the V2V communication can be expected with the adaptive modulation, shown in Table 2.

To investigate the performance of the proposed algorithm, we performed the computer simulations and compared these with the conventional static data transmission. We assume for a fixed time interval for how long the vehicle keeps trying to transmit the data to other vehicles. Also, the success rate for the connection is assumed to be 100% if there is a connectable vehicle. When there is no connectable vehicle, the data is assumed to be queued in the buffer. For the physical topology of the vehicle network, we assume that the maximum number of adjacent vehicles is 4 and the number of connectable vehicles is randomly generated from 0 to 4. Depending on the different N values, the different modulation order is determined. When N equals zero, the data is stored in the buffer without transmission in both conventional and proposed schemes. When N is not equal to zero, the conventional schemes assume the fixed modulation, such as QPSK, 16QAM, or 64QAM. In the proposed method, the different modulation type is chosen. More specifically, 64QAM is selected when N equals to 1 or 2. 16QAM is selected for N = 3 or N = 4. When N is larger than 4 in a realistic environment, the modulation order can be kept as 16QAM, as N = 4. This is because there are many possibilities that vehicles can be connected when N is larger than 4. Note that in the conventional method, 64QAM is continuously utilized during the entire time period for the ideal situation. This is because, in an 802.11p standard, 64-QAM can be reached when the channel is optimal with a line-of-sight (LOS) component and a low doppler shift [24].

Rician fading channel is assumed in the simulation. The Rician K-factor is defined as the ratio of the signal power in the dominant component over the scattered power. The K-factor is normally set to a constant value in the simulations [28]. However, reference [28] investigated the variation of the K-factors in variable vehicular channels and found that the K-factor became lower when more vehicles existed. Furthermore, reference [29] estimated the K-factor for the V2V communication and concluded that the K-factor ranged from −10 dB to 10 dB in rural, suburban, and urban environments. Therefore, we consider two scenarios, where one is scenario I with the fixed K-factor of 10 dB and another is scenario II with the different K-factors from 10, 5, −5, and −10 dB depending on the number of connectable vehicles. The turbo code with 1/2 code rate is chosen as the channel coding scheme for all conventional methods and the proposed method. Detailed simulation parameters for two simulations are summarized in Table 3.

## 4. Numerical Results

**Figure 4:**
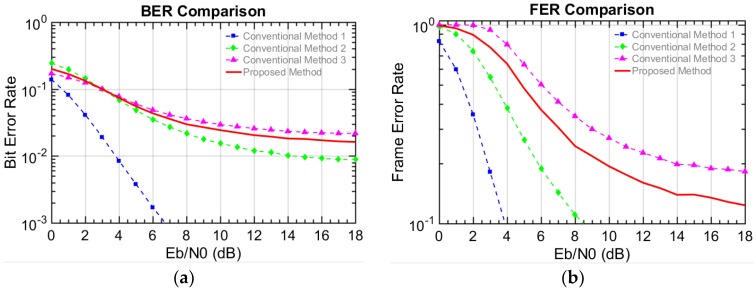
Error rate performance of V2V scenario I in a Rician fading channel a with a constant K-factor value (10 dB): the (**a**) BER performance and the (**b**) FER performance.

**Figure 5:**
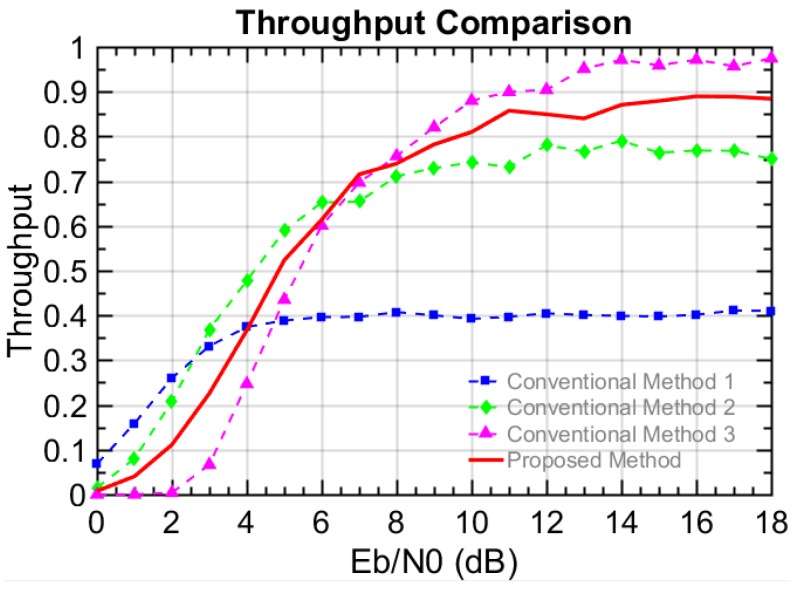
Throughput performance of V2V scenario I in a Rician fading channel with a constant K-factor value (10 dB).

**Figure 6:**
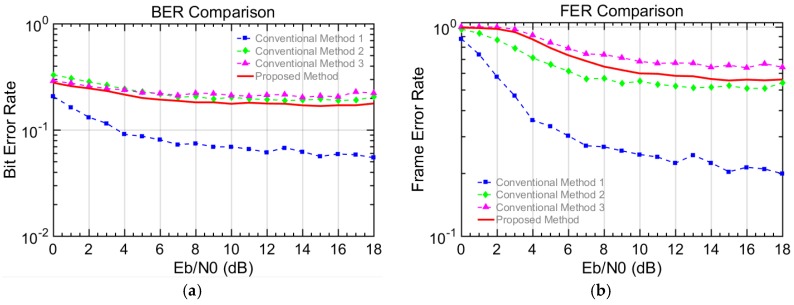
Error rate performance of V2V scenario II in a Rician fading channel with various K-factor values: the (**a**) BER performance and the (**b**) FER performance.

**Figure 7:**
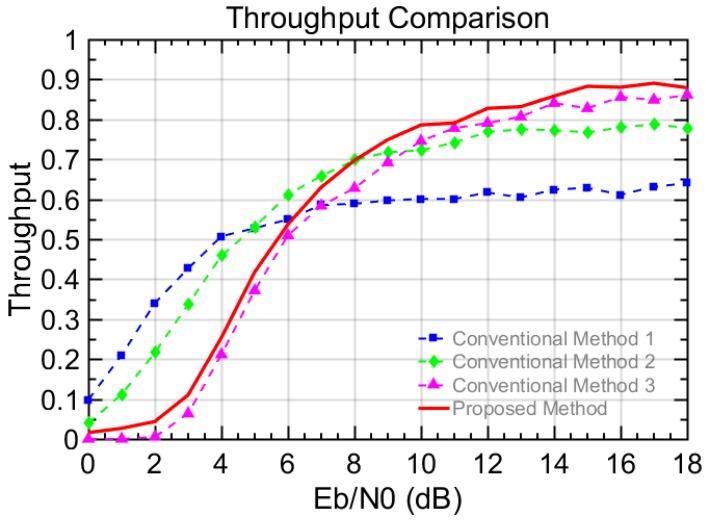
Throughput performance of V2V scenario II in a Rician fading channel with various K-factor values.

The performances are evaluated in terms of BER, frame error rate (FER), and throughput. The number of transmitted bits or frames is used for the BER and the FER, respectively. However, note that the number of total generated frames is considered for the throughput, since the generated but un-transmitted frames need to be considered. In this paper, the measure of reliability can be interpreted in terms of throughput defined in Equation (4). This is because the throughput here reflects not only the successfully transmitted bits but also the total generated bits including un-transmitted bits. Thus, the higher throughput shows the un-transmitted bits for N = 0 are compensated with the higher order modulation. Therefore, we can say the reliability gets improved in terms of the throughput. For example, a user in the vehicle can enjoy a reliable and stable stream video service, even if the vehicle cannot be connected to the other vehicles in a certain time period. The simulation results presented in Figure 4, Figure 5, Figure 6 and Figure 7, where the X axis is the energy per bit to noise power spectral density ratio (Eb/N0).

(2) $$\mathrm{BER}=\frac{\mathrm{Number}\;\mathrm{of}\;\mathrm{unsuccessfully}\;\mathrm{transmitted}\;\mathrm{bits}}{\mathrm{Number}\;\mathrm{of}\;\mathrm{transmitted}\;\mathrm{bits}},$$

(3) $$\mathrm{FER}=\frac{\mathrm{Number}\;\mathrm{of}\;\mathrm{unsuccessfully}\;\mathrm{transmitted}\;\mathrm{frames}}{\mathrm{Number}\;\mathrm{of}\;\mathrm{transmitted}\;\mathrm{frames}},$$

(4) $$\mathrm{Throughput}=\frac{\mathrm{Number}\;\mathrm{of}\;\mathrm{successfully}\;\mathrm{transmitted}\;\mathrm{frames}}{\mathrm{Number}\;\mathrm{of}\;\mathrm{total}\;\mathrm{generated}\;\mathrm{frames}},$$

Figure 4 shows the BER and FER results for scenario I. In Figure 4a, the BER of the proposed method is higher than that of conventional methods 1 and 2 but slightly lower than that of conventional method 3. Similar results were seen for the FER performance in Figure 4b. This is because the lower the modulation level is, the more robust against noise and interference it becomes. Therefore, in the conventional methods, the relatively lower level modulation shows a lower error rate. Since the proposed method randomly combines two different modulations, the error performance is also combined and located between the conventional methods 2 and 3. Note that the main objective is not the error performance but the overall performance in terms of the throughput. That is, it is more important that how much data is successfully delivered to other vehicles in terms of the throughput. In Figure 5, the throughput performances are presented. When the Eb/N0 is larger than 6.5 dB, the proposed method shows better performance than the conventional methods 1 and 2 but shows worse performance than the conventional method 3. Below 6.5 dB, the proposed scheme provides a lower throughput than the conventional method 2 but a higher throughput than the conventional method 3. Regarding conventional method 1, the maximum throughput performance is below 50%, since they transmit the data very slowly and fail to compensate for the throughput degradation when N = 0 (i.e., no connectable vehicle around). Therefore, the throughput reaches a relatively lower cap, which means the service cannot be provided adequately for this case. Even though 16QAM in conventional method 2 can bring the maximum throughput up to 76%, the throughput loss of 24% still makes the users suffer from discontinuity. On the other hand, the maximum throughput of conventional method 3 reaches near 97%. This is because 64QAM fully takes advantage of the good channel when the K-factor is fixed to 10, which is actually an ideal condition in the V2V communication. In scenario I with the ideal situation, it is shown that the proposed method cannot show its advantage over conventional method 3 but achieves 90% throughput performance, since the employment of 16QAM causes the loss of more data transmission.

Scenario II with various K-factors is more practical in comparison to scenario I with a constant K-factor. In scenario II, we set different Rician K-factor values depending on the number of connectable vehicles, in order to make the simulation closer to the realistic V2V environment. In Figure 6, the BER and FER performances are shown for conventional methods 1, 2, and 3, as well as the proposed method. In Figure 6a, the BER of the proposed method is lower than that of conventional method 2 and 3 and only higher than that of conventional method 1, which guarantees the low error rate at the expense of a lower data transmission rate. In Figure 6b, the proposed method presents a higher FER performance than conventional method 1 and 2 but a lower one than that of conventional method 3. Regarding the throughput performance in the Figure 7, the proposed method shows lower throughput than conventional methods 1 and 2 when EbN0 is smaller than 6 dB, but it exceeds that of conventional method 1 when EbN0 reaches 6 dB and exceeds that of conventional method 2 when EbN0 reaches 8 dB. The reason for this is that the error rate drops as the EbN0 is sufficiently large and, thus, higher order modulations such as 16QAM and 64QAM start to show their advantages in terms of the throughput. Also, the throughput of the proposed method is always higher than that of conventional method 3, because the relatively lower order modulation of 16QAM is used against the harsh channel situation when the K-factor is a negative value. Meanwhile, the modulation is fixed to 64QAM in conventional method 3. When EbN0 is larger than 8 dB, the proposed method shows the highest throughput, which means that the proposed scheme with the adaptive transmission may give the benefits for frequently changing topology in the VANET and, thus, improve the continuity and the reliability of the V2V communication.

## 5. Conclusions

In this paper, we proposed a dynamic transmission scheme with a variable data rate according to the number of connected vehicles and the random topology in VANET. The numerical results verified that the proposed scheme was able to provide more continuous and reliable V2V communication and to show better performance in terms of throughput compared to the conventional scheme in 802.11p. Note that the only objective is not the error performance but the throughput performance. When the Eb/N0 is larger, the proposed method generally shows much benefit compared to the conventional methods. Therefore, the proposed scheme with the adaptive transmission is proven to be generally better for the frequently changing topology and the channel environments in the VANET and, thus, may improve the continuity and the reliability of the V2V communication, even though the complexity may be rather increased. For future research, the effect of a power boost on system performance will be analyzed to further improve power efficiency. Also, the different probabilities for N = 0, 1, 2, 3, and 4 can be considered.

## Figures and Tables

**Table 1:** Receiver sensitivities for different modulation and coding types [27].

Modulation and Coding Types	Receiver Sensitivity *S_R_* (dBm)
BPSK 1/2	−99
QPSK 1/2	−96
QPSK 3/4	−94
16QAM 1/2	−90
16QAM 3/4	−87
64QAM 2/3	−82
64QAM 3/4	−80

**Table 2:** Modulation types.

Number of Connectable Vehicles, N	0	1	2	3	4
Conventional Method 1	Store data	QPSK	QPSK	QPSK	QPSK
Conventional Method 2	Store data	16QAM	16QAM	16QAM	16QAM
Conventional Method 3	Store data	64QAM	64QAM	64QAM	64QAM
Proposed Method	Store data	64QAM	64QAM	16QAM	16QAM

**Table 3:** Simulation parameters for scenarios I and II.

Rician Fading K-Factor	Number of Connectable Vehicles, N	0	1	2	3	4
	Scenario I	10 dB	10 dB	10 dB	10 dB	10 dB
	Scenario II	10 dB	10 dB	5 dB	−5 dB	−10 dB
Channel Coding Scheme	Turbo code with 1/2 coding rate					
Frame Size	1024 bits/frame					
Data Generation Speed	Scenario I	240 frames/s				
	Scenario II	120 frames/s				
Data Transmission Speed	QPSK	120 frames/s				
	16QAM	240 frames/s				
	64QAM	360 frames/s				
Simulation Time	Scenario I	1000 s				
	Scenario II	2000 s				

K is Rician Fading K-factor (dB).
